# Effects of Climate Change on Areas Suitable for Maize Cultivation and Aflatoxin Contamination in Europe

**DOI:** 10.3390/toxins15100599

**Published:** 2023-10-04

**Authors:** Marlous Focker, Michiel van Eupen, Peter Verweij, Cheng Liu, Charlotte van Haren, H. J. van der Fels-Klerx

**Affiliations:** 1 Wageningen Food Safety Research (WFSR), 6708 WB Wageningen, The Netherlands; 2 Wageningen Environmental Research (WEnR), 6708 PB Wageningen, The Netherlands; michiel.vaneupen@wur.nl (M.v.E.); charlotte.vanharen@wur.nl (C.v.H.)

**Keywords:** iCLUE, land use, mycotoxins, modeling, climate change, temperature

## Abstract

The climate is changing in Europe: average temperatures are increasing, and so is the frequency of extreme weather events. Climate change has a severe impact on areas suitable for growing certain crops and on food safety, for example, affecting the occurrence of the aflatoxin contamination of maize. The aim of this study was to obtain insights into the impact of climate change on possible changes in land use in Europe, particularly in areas suitable for maize cultivation, and on the probability of the mycotoxin contamination of maize in order to give directions for long-term adaptation to climate change. By combining a land use model and a mycotoxin prediction model, the suitability of land for maize cultivation and the probability of aflatoxin contamination were estimated for suitable areas in Europe, comparing the current climate with the 2050 scenario. In 2050, the occurrence of aflatoxin contamination in Europe is predicted to severely increase, especially in Central and Southern Europe. More northern regions, presently unsuitable for maize cultivation, will become suitable for maize cultivation in 2050. In the baseline scenario, most regions suitable for maize cultivation have a low probability of aflatoxin contamination, whereas in 2050, about half of the regions suitable for maize cultivation have a medium to high probability of aflatoxin contamination. Regions for safely growing maize for human consumption will shift from the southern to the northern half of Europe.

## 1. Introduction

Since the green revolution in the mid-1950s, cereal production in Europe has increased drastically [1]. At the same time, the climate has been changing: average temperatures are increasing, precipitation patterns are changing, the CO_2_ level is increasing, and the frequency of extreme weather, such as droughts, flooding, or storms, is increasing [2]. Temperatures are predicted to rise in Europe at a rate exceeding global mean temperature changes [2]. In addition, hot extremes will be more frequent and intense [2,3,4,5]. For Northern, Western, Central, and Eastern Europe, increased precipitation and potential floods are expected at a global warming temperature of 1.5 °C and above. For Northern Europe, severe windstorms are expected at a global warming temperature of 2 °C and above. For Western and Central Europe and for the Mediterranean region, hydrological, agricultural, and ecological droughts are expected at mid-century-predicted warming levels of 2 °C and above [2]. 

These changes in our climate have consequences for European agriculture and food safety [6,7]. The first consequence of climate change relates to land use. Land use changes due to climate, human interventions, and natural disasters [8]. Some areas may no longer be suitable for the cultivation of certain crops due to heat or drought, whereas new areas might become suitable for growing certain crops due to the higher temperatures in these areas [9]. In addition, climate change may also result in structural changes, such as floods that damage or wash away existing land [10,11]. Finally, in the future, new agricultural land may be gained or existing land may be lost for reasons other than climate change, for example, due to deforestation or the transformation of fertile land for urban extension, respectively [8]. 

In addition to changes in land use, climate change leads to changes in the presence of food safety hazards. One main category of food safety hazards expected to be impacted by climate change is that made up by mycotoxins [12]. Mycotoxins are toxins produced by certain fungal species after the infection of crops. Mycotoxins are known to have toxic effects on humans and animals and are therefore unwanted in feed and food [13]. Since both the infection of crops by fungi and the production of mycotoxins are largely governed by weather, climate change is expected to have a large impact on the mycotoxin contamination of crops [14]. Aflatoxins are genotoxic, carcinogenic, and immunotoxic, and they are considered to be one of the most toxic groups of mycotoxins [15] Aflatoxins can lead to severe consequences in farmed animals and humans [16]. Aflatoxins are produced by *Aspergillus* spp., particularly *A. flavus*, infecting, amongst other crops, *Zea mays* (maize). *Aspergillus* spp. are thermotolerant fungal species that are adapted to warmer climates. As a consequence, aflatoxins mainly occur in crops such as maize, rice, and nuts, which are grown in tropical and subtropical areas [17]. However, with climate change, it has already been predicted by several studies that aflatoxins in maize will also become a main food safety hazard in Europe. Weather extremes such as droughts and high temperatures are main drivers of aflatoxin production [18,19,20]. Today, *A. flavus* as well as aflatoxins have been observed in high concentrations in Italy, Croatia, Serbia, and Hungary, amongst other Southern European countries [6]. In 2012, hot and dry weather conditions in Serbia led to high mold growth and aflatoxin production in maize. This highly contaminated maize was fed to dairy cattle, leading to high aflatoxin levels in Serbian milk in 2013 and 2014, consequently leading to a severe economic impact [21]. 

To improve food safety management, there is a need for the prediction and timely recognition of food safety hazards. Mycotoxin prediction models can give directions for long-term adaptation to climate change [22]. It might, for instance, be decided to grow alternative crops in regions with high levels of mycotoxins year after year. Mycotoxin prediction models predicting the presence of aflatoxins in maize (for example, PREMA) are currently available [23]. Furthermore, by linking mycotoxin prediction models to land use models, climate change scenarios can be investigated, and directions can be given for safe crop cultivation [24]. Land use models, such as iCLUE, that can predict where the land use changes are likely to take place are already available as well [8]. This study aimed to combine the PREMA and iCLUE models to obtain insights into the possible changes in land use in Europe, particularly in areas suitable for maize cultivation, and the probability of mycotoxin contamination of this maize. Two climate scenarios were compared for Europe: the current climate and a 2050 scenario.

## 2. Results

### 2.1. Output of the iCLUE Model

**Figure 1:**
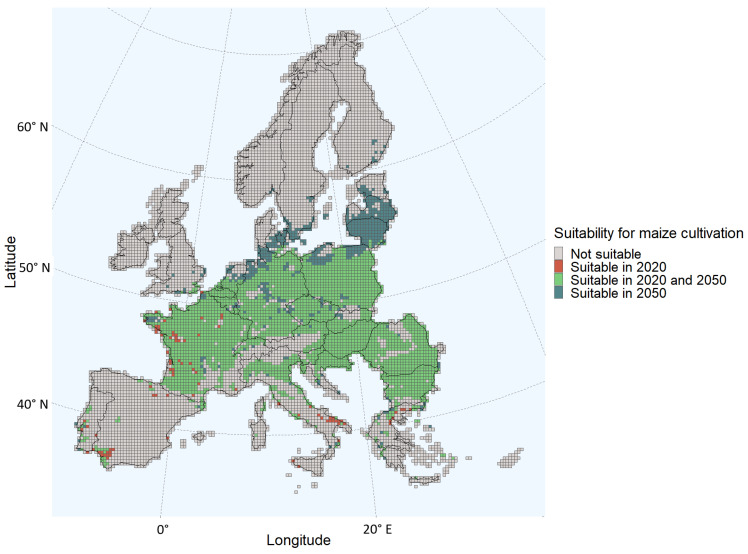
Changes in suitability for maize cultivation for each grid of the European Union Member States and/or regions in the Schengen area.

The grids suitable for maize cultivation in the present scenario are shown in Figure 1. The 2050 scenario showed that under the climate scenario considered in this study, more grids in Europe will become suitable for maize cultivation, with an increase of 16% compared to 2020. Furthermore, the areas suitable for maize cultivation are more northerly located in Europe compared to the current scenario. Some areas in France, Spain, Italy, and Greece that are suitable for maize cultivation in the present scenario will no longer be suitable according to the 2050 scenario, whereas more areas in Northern Europe, such as most of Latvia and Lithuania, are estimated to be suitable for maize cultivation in 2050. Even a few grids in the South of Sweden, Denmark, Estonia, and Finland are classified as suitable for maize cultivation under the 2050 scenario (Figure 1).

### 2.2. Output of the PREMA Model

**Figure 2:**
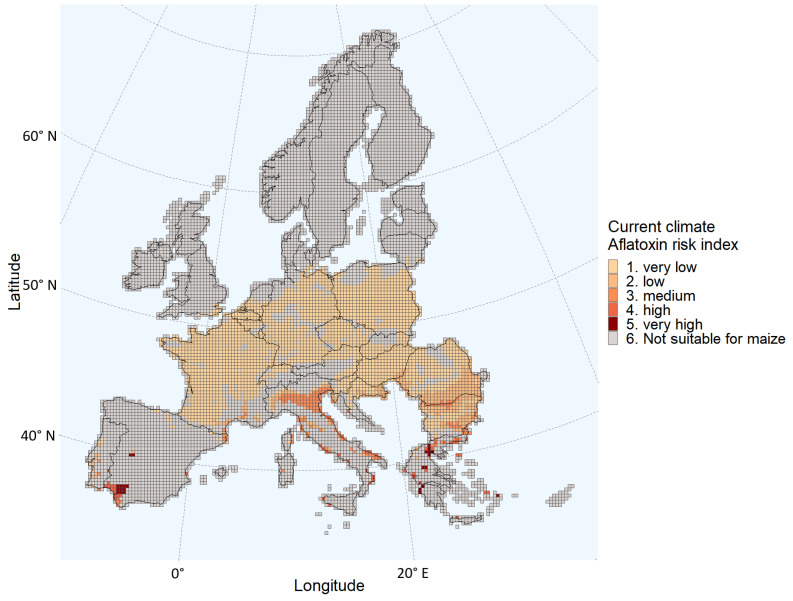
Aflatoxin Risk Index estimated for each grid of the European Union Member States and/or regions in the Schengen area under the present climate conditions.

The outputs from the iCLUE and PREMA models are summarized in Figure 2 and Table 1. In the present scenario, the regions that are expected to be largely suitable for maize cultivation and have a very-low to medium probability of aflatoxin contamination are France, Belgium, the South of the Netherlands, Germany, Poland, Czech Republic, Slovakia, Hungary, Romania, and Slovenia (Figure 2). Certain areas of Bulgaria, Italy, Portugal, and Croatia have an estimated medium probability of aflatoxin contamination. The grids in Spain and Greece were largely classified as not suitable for maize cultivation. In addition, the few grids suitable for maize cultivation in these two countries had an estimated medium to high probability of aflatoxin contamination (Figure 2). For the current climate conditions, the vast majority of grids (93%) were classified as having a “very low” or “low” probability of aflatoxin contamination. The few grids classified as having a “high” or “very high” probability were equal to 2% of all the grids suitable for maize cultivation (Table 1).

**Figure 3:**
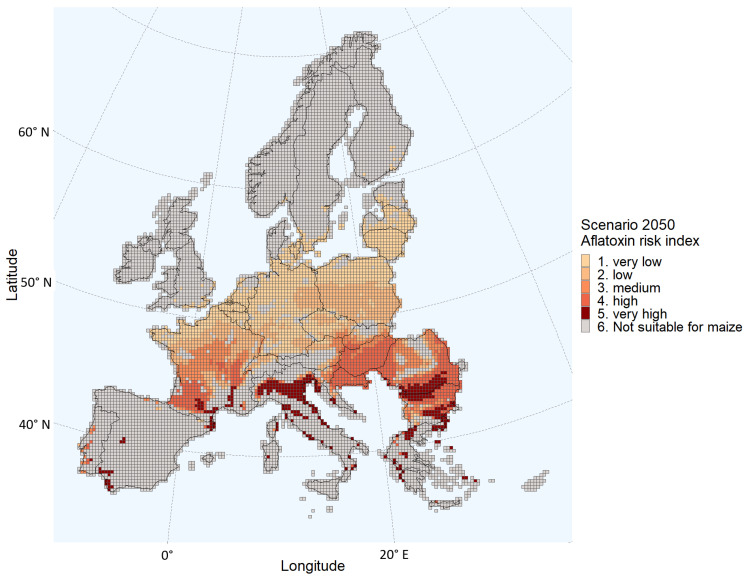
Aflatoxin Risk Index estimated for each grid of the European Union Member States and/or regions in the Schengen area under the 2050 climate scenario.

**Figure 4:**
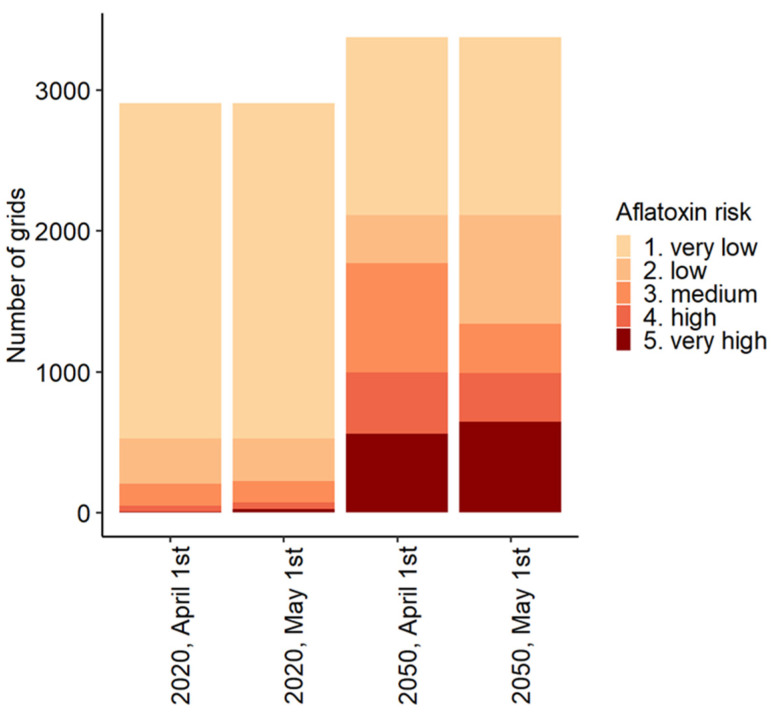
Number of grids in Europe per Aflatoxin Risk Index (ARI) category for the present and 2050 climate scenarios, with planting dates fixed at 1 April or 1 May.

The calculated growing seasons for both the current climate and the 2050 scenario shows that with a fixed planting date, the dates of emergence, flowering, and maturity will be earlier in the season in the 2050 climate change scenario (Table 2). More grids are estimated to have an elevated (medium to very high) probability of aflatoxin contamination in the 2050 scenario compared to the present scenario (Table 1 and Figure 3). In the present scenario, 93% of the total grids suitable for maize cultivation have a very low or low probability of aflatoxin contamination, whereas in the 2050 scenario, only 47% of the grids suitable for maize cultivation have a very low or low probability of aflatoxin contamination (Figure 4). Grids with an elevated aflatoxin contamination probability are located farther north in the 2050 scenario compared to the current climate scenario. For example, several grids in Central France showed an elevated ARI in 2050, whereas only a very few grids in Southern France had a medium probability of aflatoxin contamination in the present scenario. In the 2050 scenario, 562 grids are classified as having a “very high” probability; in contrast, 5 grids are classified as such in the present scenario. These 562 grids of the 2050 scenario are located in Spain, Portugal, the South of France, Italy, the south of Bulgaria, Romania, and the south of Croatia and Greece.

Figure 4 depicts the sensitivity of the model to the chosen planting dates. When the planting date was set to 1 May instead of 1 April, the probability of aflatoxin contamination generally increased. Under the current climate scenario, the number of grids in the categories “very low”, “low”, and “medium” probability decrease with a later planting date (0.9%), whereas the number of grids with the categories “high” and “very high” increase with a later planting date (50%). For the 2050 scenario, similar results were observed, although the differences are smaller. With a later planting date, the number of grids in the categories “very low”, “low”, and “medium” probability decrease (0.3%), whereas the number of grids with the categories “high” and “very high” increase with a later planting date (9.5%).

Concentrations of AFB1 in maize are summarized in Table 3; they are based on the yearly mycotoxin survey from DSM. Using the DSM classification, data are presented for Northern, Central, and Southern Europe. For Northern Europe, there were no data for aflatoxins in maize between 2017 and 2020; this outcome was expected since these countries are not suitable for maize cultivation due to the current climatic conditions. Between 2017 and 2020, on average, 13% of the maize samples collected in Central Europe were above the detection limit of 2 µg/kg; these (positive) samples had an average aflatoxin concentration of 7 µg/kg. In Southern Europe, on average 20% of the samples collected exceeded the detection limits; these samples had an average concentration of 5 µg/kg. Monitoring data showed that 13% of the samples collected from countries in Central Europe between 2017 and 2020 had aflatoxin levels above the detection limit, which could match the very low to medium aflatoxin contamination probability classes. Monitoring data showed that 20% of the samples collected between 2017 and 2020 in Southern Europe had aflatoxin concentrations above the detection limit, matching, in our opinion, the classes medium to high probability.

### 2.3. Comparing the Change in Aflatoxin Risk Index (ARI) and the Change in Temperature

**Figure 5:**
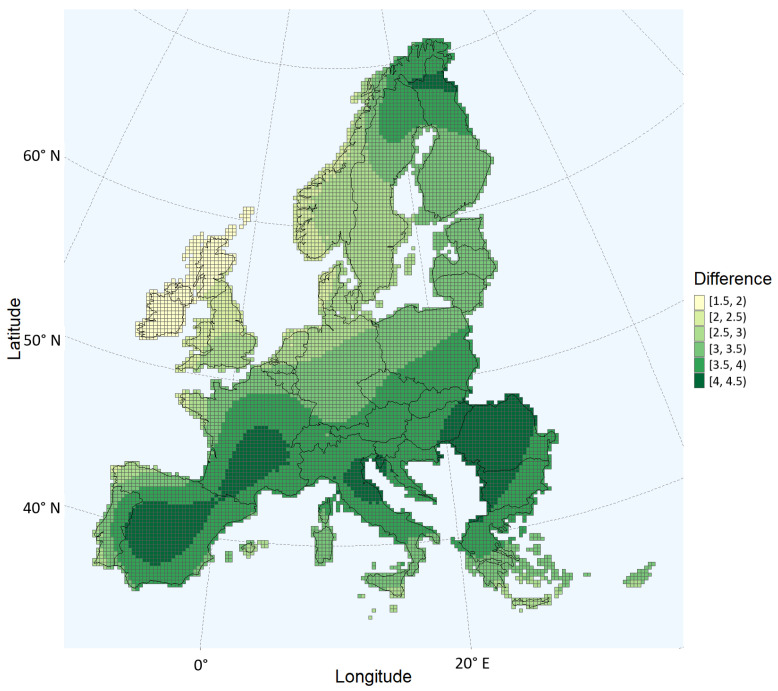
Change in average daily maximum temperatures, in degrees Celsius, between the current climate and the 2050 scenario. The months from April to October, the maize growing period, are considered.

Comparing Figure 1 and Figure 3, the probability of aflatoxin contamination in Central and Southern France and in Italy, Hungary, Croatia, Romania, and Bulgaria seems to increase drastically in the 2050 scenario relative to the current climate. The estimates for these regions coincide with the regions having the largest changes in daily maximum temperatures during the growing season (Figure 5). For example, regions in the south of Romania having an estimated low probability in the current climate scenario are estimated to have a very high probability in 2050. These are regions with a maximum temperature change between 4 and 4.5 °C during the growing season.

## 3. Discussion

In this study, first, the suitability of regions in Europe for maize cultivation was predicted given expected climate change effects. The probability of mycotoxin contamination of maize was then predicted only for those grids suitable for maize cultivation. By linking mycotoxin prediction models to land use models, climate change scenarios can be investigated and directions can be given for safe crop cultivation in the future [24]. Until today, studies dealing with predicting the probability of aflatoxin contamination under the influence of climate change have not considered the suitability of land for the cultivation of specific crops [19,29]. In this respect, this is the first study that combines a land use model with a mycotoxin prediction model. The current study was able to estimate both the areas suitable for maize cultivation and the probability of aflatoxin contamination. This combined information is helpful in choosing the crops to be grown in these areas and the end use of the resulting produce (e.g., human consumption, animal feed, or biofuel).

Adding land use models to predictive models for mycotoxin contamination can help to target control options to areas most suitable for crop cultivation with a high probability of mycotoxin contamination. In 2001, the European Commission adopted Commission Regulation 2001/466/EC, setting the maximum limits for the presence of contaminants in food products, including maximum limits for concentrations of aflatoxins in diverse food products [30]. Given the compliance with these legislative limits for food, the risk related to the exposure of the European population to aflatoxins via food consumption is limited [31]. Even though maximum limits for aflatoxins in food for human consumption as well as animal feed are in place in the European Union, more prevention and control measures, including sampling and analyses, are and will be needed to manage the aflatoxin levels in food and feed products as climate change continues [32,33]. Climate change will result in generally higher levels of aflatoxins and a higher number of batches that do not satisfy the maximum limits set. Without prevention and control measures prior to monitoring, a high number of batches would need to be discarded, leading to high economic losses. The highest burden is on maize growers due to the rejection of batches with high levels of aflatoxins [33]. Several prevention and control options are being investigated both at pre-harvest and at post-harvest levels. Since the impact of the control options is strongly influenced by the degree of mycotoxin contamination and the volumes produced in a certain area, predictive models for mycotoxin contamination can help in determining the most cost-effective prevention and control options for specific regions [34]. 

From the present to 2050, the climate is expected to change, and so is land use and the probability of mycotoxin contamination. In 2050, the probability of aflatoxin contamination in Europe is predicted to increase, especially in Southern Europe. More northern regions, previously not suitable for maize cultivation, will become suitable for maize cultivation with increasing temperatures towards 2050. Based on our results, it seems that in the future, maize grown in some areas of Southern Europe might become unsuitable for human consumption. Several papers have discussed the presence of mycotoxins in relation to climate change or predicted regions at risk for elevated mycotoxin levels under different climate scenarios [19,29,33]. In the study conducted by Battilani et al. (2016) [19], the same trend reported in the current study was observed, namely, the ARI will increase over time with climate change. Areas with a high probability of aflatoxin contamination are currently observed in the South of Spain, Italy, and Greece and are moving north up to the South of France, the north of Italy, and the south of Romania, where an increase in temperature of a few degrees Celsius is predicted in the next few decades. Yu et al. (2022) [33] estimated a similar trend in the United States. Aflatoxin events in maize currently confined to states in the south are predicted to shift toward the Corn Belt, located farther north.

Even though this study provides useful information and stresses again the urgency of the aflatoxin problem Europe will soon face, it has a number of limitations. First of all, monthly weather data were used in our model instead of daily weather data. Even though the use of monthly weather data is a limitation of this model, future daily weather data are extremely uncertain and, if available, often the result of postprocessing monthly data [35,36]. Therefore, in our opinion, the use of daily weather data would not have significantly improved our results. Second, we were not able to establish a direct relation between the aflatoxin risk index (ARI) and mycotoxin contamination due to a lack of access to non-summarized monitoring data in the study areas. The risk indexes were classified based on expert knowledge and compared with the current aflatoxin contamination distribution (Table 4). The distribution shown in Figure 2 is generally in line with current observations (Table 4) and therefore validated the classification threshold in this study to a certain extent. European monitoring data on aflatoxin in maize must be acquired along with detailed locations and sampling dates in order to further validate our results. Predictions of future mycotoxin concentrations could also be used in order to predict economic losses or increased toxic effects. A third limitation of this study is that the model used to estimate the ARI does not encompass all steps of the *A. flavus* infection cycle. For example, dispersal is dependent on precipitation and relative humidity data, which are not included in our model due to the lack of daily precipitation data available when using climate change models. In addition, no other drought indicator could be included due to the uncertainty of daily precipitation predictions for the 2050 scenario. Precipitation and relative humidity are known to have an effect on the dispersal of *A. flavus* spores. There is no spreading of spores on rainy days or when the relative humidity is higher than 80% [37]. Water activity has an effect on sporulation, although it is often not a limiting factor [38]. Due to the lack of daily data regarding these factors, it was not possible to include these effects in the current model. Chauhan et al. (2015) [38] included drought as a relevant factor instead of relative humidity or precipitation. In the case of drought, crops are more susceptible to fungal infections. Precipitation and irrigation or a drought index are factors that should be added to an improved version of this model in the future. This would require the collection of more data throughout Europe. Fourth, there is still a need to investigate the combined effect of multiple climate change effects, e.g., periods of drought, flooding, increased temperature, or storms, on the growth of crops, the growth and infection cycle of mycotoxin-producing fungi, and the production of mycotoxins. Fifth, pest damage has a strong influence on the probability of aflatoxin contamination [39]. This factor was also not considered in the current model and should be added to an improved version of the model. Data on pest damage in multiple European countries should also be collected in the future. A sixth assumption made is that Aspergillus spp. were present in the environment for all grids. No data were available to validate this assumption; this could also be improved by measuring the presence of different types of fungi in multiple European countries. Seventh, in our model, the planting dates were fixed. The effect of the planting date was found to be limited for the current climate but had a larger effect in the 2050 scenario. To account for the shift in the growing season in the future, more-advanced crop phenology models or a similar approach to that taken by Yu et al. (2022) [33], who modelled future growing seasons as a function of projected weather conditions to maximize yield, could be used. 

To conclude, with the expected climate change, the probability of aflatoxin contamination in maize is expected to rise drastically in Southern and Central Europe. Suitable regions for safely growing maize for human consumption will shift from the southern to the northern half of Europe.

## 4. Materials and Methods

### 4.1. Climate Scenarios

In predictive studies, scenarios are used to describe how the socio-economy and the climate may develop. The climate scenario considered in this study is the ‘intermediate’ Representative Concentration Pathway (RCP 4.5) [40], which assumes a mean global temperature rise of 1.8 °C, 0.47 m average sea level rise, and a moderate increase in extreme weather events by 2100 as a result of changing greenhouse gas concentrations. Although the mean global temperature is projected at 1.8 °C, strong regional differences are to be expected. Dunne et al. (2012) [41] captured the scenario’s assumptions in the GFDL ESM2 global coupled climate–carbon earth system model to project monthly weather data with 0.5 by 0.5° spatial resolution. Future daily weather data are extremely uncertain; therefore, climate models most often project monthly weather data. Daily weather models are often based on monthly estimations. For this reason, monthly climate change data were used in this study.

### 4.2. iCLUE Model

The iCLUE model is a land use model that predicts where land use changes are likely to take place; this model is part of the CLUE model family [42,43,44,45]. iCLUE allocates land use based on (i) the areal demand for every land use class (e.g., a certain number of hectares for agriculture), (ii) land use suitability (e.g., dry unfertile soil is not suitable for agriculture), (iii) neighboring land use (urban areas are likely to expand to neighboring land), and (iv) conversion rules (newly planted forests are only harvested after they have fully grown). In this study, areal demands were derived from FAO trend extrapolation, modelled macro-economic projections, and Copernicus landcover statistics [46], following the Shared Socio-economic Pathway ‘middle of the road’ (SSP2) [47].

The PREMA model, described in the next section, uses 25 by 25 km grids. Therefore, the 0.5 by 0.5° spatial resolution grids generated by the climate and iClue models were aggregated to larger 25 by 25 km grids. The suitability for maize cultivation of each 25 by 25 km grid was estimated for countries belonging to the European Union, the Schengen area, or the United Kingdom by combining the agro-climatic crop suitability [41,48] with the allocated cropland areas determined by iCLUE. This model generated, for the present and 2050 climate scenarios, the square kilometers within a grid suitable for maize cultivation, the square kilometers suitable for other crops, and the square kilometers suitable for non-cropping areas. All grids with at least 25 km^2^ suitable for maize cultivation (>4%) and having more square kilometers suitable than unsuitable for maize cultivation in a particular grid were considered in estimating the presence of aflatoxins.

### 4.3. PREMA Model

In addition to the estimation of the suitability for maize cultivation of each 25 by 25 km grid, the probability of the formation of aflatoxins B1 (AFB1) in maize was estimated for the relevant grids. Even though land can be suitable for maize cultivation, if the predicted level of aflatoxin contamination is too high, the area could be considered unsuitable for maize cultivation. 

To predict the presence of aflatoxins in maize, an adapted version of the PREMA model was used [23]. The mechanistic part of this model, including the growth of fungi and the production of aflatoxins, is derived from the AFLA-maize model developed by Battilani et al. (2013) [37]. Since this model uses daily weather data as inputs, and as our study used monthly data, daily weather data were artificially created, keeping the minimum, maximum, and average temperatures of the climate model data constant throughout the month. The susceptible period for *Aspergillus* spp. growth and formation of aflatoxins was considered to be between the day of emergence of the maize crop and the day of harvest. For both the present and 2050 scenarios, the planting date was assumed and fixed at 1 April for all grids [23]. As a sensitivity analysis, the model was also run with a planting date fixed at 1 May. For both scenarios, the full maturation date was used as an approximation of the harvest date. The dates of emergence, flowering, and harvest were estimated based on the planting date and the growing degree days: 50 growing degree-days (Celsius) were needed from planting to emergence, 750 growing degree days were needed from emerging to flowering, and 750 growing degree-days were required from flowering to harvest [37,49]. Risk scores (without unit) for *A. flavus* growth and aflatoxin production for this period were estimated using the method described by Battilani et al. (2013) [37] and Liu et al. (2021) [23] with the following equations:

(1) $$GROWTH,AFLA={\left[A\times {Teq}^{B}\times \left(1-Teq\right)\right]}^{C}$$

(2) $$Teq=\frac{T-Tmin}{Tmax-Tmin}$$

Above, A, B, C, Tmin, and Tmax are constant parameters listed in Table 4, and T is the average temperature of the day.

**Table 4:** Input parameters to estimate the growth of *A. flavus* and the production of aflatoxins. These parameters and their values come from the study of Battilani et al. (2013) [37].

	GROWTH	AFLA
A	5.98	4.84
B	1.70	1.32
C	1.43	5.59
Tmin (in °C)	5	10
Tmax (in °C)	48	47

Both *A. flavus* growth and aflatoxin production are a function of the temperature and water activity inside a kernel. The water activity inside a kernel is considered to be favorable starting from silk emergence [37]; therefore, growth and aflatoxin production were estimated from this stage onwards. 

Prior to growth and aflatoxin production, *A. flavus* needs to sporulate and germinate. These two steps are both influenced by temperature and water activity. The equations used to estimate this risk score can be found in the research conducted by Battilani et al. (2013) [37]. It was assumed that *A. flavus* was present in the environment for all grids, a precondition for sporulation [37]. Dispersal is influenced by rain or high humidity. On rainy days or very humid days (>80% relative humidity), it was assumed that there would be no dispersal [37]. However, using future weather data, it was impossible to predict with reasonable certainty which days we could expect rain. Hence, dispersal was considered a non-limiting step, similar to the model of Chauhan et al. (2015) [38].

The aflatoxin risk index (ARI) was estimated by multiplying the risk scores for each step: *A. flavus* sporulation, germination, growth, and aflatoxin production. The ARI was estimated for grids that were suitable for maize cultivation only, according to the output of the iCLUE model. The ARI was expressed in 5 classes: very low (ARI < 10), low (10 ≤ ARI < 20), medium (20 ≤ ARI < 30), high (30 ≤ ARI < 40), and very high (ARI ≥ 40). This risk index could not be directly translated to mycotoxin concentrations since mycotoxin concentration data for specific regions in Europe were unavailable for this study. The higher the ARI, the higher the probability of aflatoxin contamination. We validated whether the classes estimated for the present scenario were realistic using aflatoxin-monitoring data published by DSM. DSM performs yearly mycotoxin surveys, monitoring mycotoxin levels, including aflatoxins, in many countries, including those in Europe [25,26,27,28].

### 4.4. Comparing the Change in Aflatoxin Risk Index (ARI) and the Change in Temperature

To illustrate the relationship between the change in temperature and the probability of aflatoxin contamination, a temperature map was created. The average maximal temperatures of the days in the growing season, from April to October, were calculated for each 25 by 25 km grid. Next, the difference between the 2050 and the 2020 scenarios was calculated.

The statistical software R, version 4.1.0 [50], was used to run the PREMA model, link the output of the iCLUE model to the output of the PREMA model, and plot all figures. The figures were plotted using several R packages: sf [51], rnaturalearth [52], rmapshaper [53], rworldmap [54], and ggplot2 [55].

## Figures and Tables

**Table 1:** Number of grids per Aflatoxin Risk Index (ARI) category for the present and 2050 climate scenarios, with a planting date fixed at 1 April.

ARI	1. Very Low	2. Low	3. Medium	4. High	5. Very High	Total
2020	2375	321	157	45	5	2903
2050	1263	341	774	432	562	3372

**Table 2:** Estimated growing season based on the daily temperatures for both the 2020 and 2050 scenarios (mean (5th–95th percentiles)).

	Assumed Planting Date	Estimated Emergence Date	Estimated Flowering Date	Estimated Harvest Date
2020	1 April	25 April (13 April –7 May)	8 August (5 July–12 September)	9 November (26 August–30 November)
2050	1 April	18 April (9 April–3 May)	18 July (22 June–10 August)	4 October (9 August–30 November)

**Table 3:** Data from DSM mycotoxin surveys related to aflatoxins in maize (aggregated data from 2017 to 2020 [25,26,27,28]).

	Positive Samples (%)					Average Concentration in Positive Samples (µg/kg)				
	2017	2018	2019	2020	Mean	2017	2018	2019	2020	Mean
Europe	22	na	na	10	16	12	na	na	12	12
Central Europe	23	15	6	8	13	na	na	7	na	7
Southern Europe	22	21	22	16	20	na	na	5	na	5

na: not available. Northern Europe: United Kingdom, Norway, Sweden, Finland, Denmark, Estonia, Latonia, and Lithuania (no maize samples in 2017–2020). Central Europe: the Netherlands, Belgium, Luxemburg, Germany, Poland, France, Switzerland, Austria, the Czech Republic, Slovakia, Slovenia, Bulgaria, Romania. Southern Europe: Portugal, Spain, Italy, Croatia, Republic of Serbia, Greece, Albania, Macedonia, Bulgaria, Bosnia and Herzegovina, Montenegro, and Turkey.

## Data Availability

The data presented in this study are available on request from the corresponding author.
